# Cardiology Training in Brazil and Developed Countries: Some Ideas for Improvement

**DOI:** 10.5935/abc.20190212

**Published:** 2019-10

**Authors:** Lucas Colombo Godoy, Michael E. Farkouh, Isabela C. K. Abud Manta, Talia F. Dalçóquio, Remo Holanda de Mendonça Furtado, Eric H. C. Yu, Carlos Gun, José Carlos Nicolau

**Affiliations:** 1 Instituto do Coração (InCor), Hospital das Clínicas HCFMUSP, Faculdade de Medicina, Universidade de São Paulo, São Paulo, SP - Brazil; 2 Peter Munk Cardiac Centre and Heart and Stroke Richard Lewar Centre, University of Toronto, Toronto, ON - Canada; 3 Brigham and Women´s Hospital, Harvard Medical School, Boston, MA - USA; 4 Instituto Dante Pazzanese de Cardiologia, São Paulo, SP - Brazil

**Keywords:** Cardiology, Education, Medical, Program Accreditation, Internship and Residency, Fellowships and Scholarships, Brazil

## Abstract

Huge variations exist in cardiology training programs across the world. In developing (middle-income) countries, such as Brazil, to find the right balance between training improvements and social and economic conditions of the country may be a difficult task. Adding more training years or different mandatory rotations, for instance, may be costly and not have an immediate direct impact on enhancing patient care or public health. In this text, we compare the Brazilian cardiology training system with other proposals implemented in developed countries from North America and Europe, aiming to point out issues worth of future discussion. Factors such as training rotations and competencies, and program duration and distribution across the countries are presented. The number of first year cardiology trainees per inhabitants is similar between Brazil and the United States (0.24 medical residents/100,000 inhabitants in Brazil and 0.26 medical residents/100,000 inhabitants in the USA). These numbers should be analyzed considering the inequality in training program distribution across Brazil, since most centers are located in the Southeast and South regions. Having more residency programs in distant areas could improve cardiovascular care in these areas. Duration of cardiology Residency Training is shorter in Brazil (two years) in comparison with developed countries (> 3 years). Brazilian residency programs give less emphasis to scientific research and diagnostic methods. Unifying minimum training requirements across the globe would facilitate the development of international learning opportunities and even professional exchange around the world.

## Introduction

Brazil is the fifth largest country in the world, regarding both land territory and population, and it is the tenth largest economy globally.^1,2^ As well as in many other developing and developed nations, cardiovascular diseases are a major concern in Brazil, being the leading cause of death in the country.^3^ On the other hand, many Brazilian citizens do not receive proper cardiovascular care.^4^

Improving health professionals’ training may contribute to change this scenario, although the understanding of what modifications should be made in cardiology training programs is not an easy task. Drawing parallels between the cardiology residency programs in Brazil and other countries’ programs may help to identify possible targets for improvement. However, there are few publications currently available on cardiology training programs.^5^ The main objectives of this report were 1) to delineate a non-systematic narrative point-of-view, comparing the Brazilian training system with countries in Europe and North America; 2) to briefly introduce the cardiology training program in Brazil to international readers who may not be familiar with it. PubMed database was searched for articles published up to February 2018 with the keywords “cardiology Residency” and “cardiology Training” in the target countries, written in English, Spanish and Portuguese. Official position statements and legal documents issued by cardiovascular societies and public organizations pertinent to this topic were also reviewed and cited as appropriate. Selection of countries was made based on the availability of information in the literature. Given the nature of the present article, and the scarcity of published data, authors’ opinions were also included when appropriate.

## Length of Training

To become a cardiologist in Brazil, the first step is to obtain a medical degree. After finishing high school, the student must apply for a highly competitive entrance exam (called “vestibular”) and be admitted to a medical degree course, which lasts six years. The following step is a two-year internal medicine residency and, finally, the general cardiology residency (named Fellowship in some countries, such as the USA), which lasts two more years. To be a specialist in a field of cardiology, additional training is required. For example, for training in imaging techniques (echocardiography, cardiac magnetic resonance, nuclear medicine, etc.), usually one or two additional years are necessary for each track. The same amount of time is normally required for some clinical subspecialties, such as Acute Coronary Care, and two years are mandatory for Interventional cardiology. For Heart Failure and Transplantation, one extra year is required. In summary, to become a Cardiologist in Brazil one needs to complete four (General Cardiologist) to six (General Cardiologist with a subspecialty training) years of postgraduate medical education. It is important to note that the National Board of Medical Residency (“*Comissão Nacional de Residência Médica*” - CNRM) supervises the residency training (as will be discussed later) and some (but not all) of the subspecialties training programs.

When comparing education and training lengths between developed and developing countries, important differences are observed (Table 1). Ten years are required to become a general cardiologist in Brazil (which is similar to most countries in Latin America).^6-9^ In the USA, internal medicine residency and cardiology Fellowship usually last three years each. Adding that to the period in the undergraduate degree program and in Medical School (eight years), the total post-secondary education time to become a cardiologist in the USA is 14 years. In Germany, three years of internal medicine residency training and three years of cardiology residency training are required (12 years in total). There are also differences in patterns of work shifts across the countries, which may influence the real time spent in training. Post-call day-off, for example, is a common practice in the USA and Canada, while in Brazil the resident is allowed six hours of rest (instead of the whole day) following a night-shift work. After the completion of the whole educational program straightly, that is, without interruptions for conscription, or extra or sabbatical years, a doctor will be able to become a cardiologist approximately at the age of 28 in Brazil, 33 in the USA and 31 in Germany.^10,11^

**Table 1:** Length of cardiology residency trainings in selected countries.^10-12,21,25,33,34^

Country	Continent	Graduation (years)	Previous training length (ys) (e.g.: internal medicine)	Cardiology residency program (years)	Total length (years)
Australia	Oceania	6	4	3	13
Brazil	South Am.	6	2	2	10
Canada	North Am.	7 or 8	3	3	13 or 14
France	Europe	6	1	3	10
Germany	Europe	6	3	3	12
Spain	Europe	6	1	4	11
UK	Europe	5	4 or 5	5	14 or 15
USA	North Am.	8	3	3	14

Am.: America. Data obtained from each country's official legislation or decree on medical training and/or National Cardiovascular Society

Another example of a lengthy training process can be observed in the United Kingdom. After finishing medical school (usually five years), the trainee must complete a two-year Foundation Program, followed by the Core Medical Training (two years) or Acute Care Common Stem (three years). After that, the trainee is finally able to go through the specialty training in cardiology. That is comprised of three initials years of Core Cardiology Training and two years of advanced training in specialist area modules. During the three initial years, the emphasis is given to acute cardiovascular care and basic procedural techniques. For the last two years, most of the time is spent in one or more of these fields of practice: interventional cardiology, electrophysiology, non-invasive imaging, adult congenital heart disease or heart failure. Counting all those years together, a total of 14 or 15 years is required to become a cardiologist in the UK. Of note, after the completion of this pathway, not all cardiologists will have the same amount of knowledge in every area, since the curriculum for the last years is flexible to the individual’s interest. Additional years may be necessary for those doing part-time training, or for dual certification in cardiology and internal medicine, or for combining out-of-program research (by doing a research fellowship in another institution, for example) or extra training in subspecialties of cardiovascular care.^12,13^

Clearly, the duration of training to become a cardiologist is not uniform across countries, and there is not a definitive standard of practice. It depends not only on the total amount of knowledge and practical skills that the professional needs to acquire, but also on the country’s social and economic conditions, since having more training years increases educational expenses. As a general guidance, the American College of Cardiology (ACC) endorses the period of three years for training in general cardiology while the European Society of Cardiology recommends a four-year term, despite important variations among European countries.^14,15^ Using a comparative approach only, it is not possible to accurately state that the same length of training in these countries would be applicable to the Brazilian reality. It may be the case that medical residents in Brazil spend more time in service during the two-year residency and oversee more patients. Conversely, the amount of knowledge in cardiology has increased dramatically in the last decades^16^ and it seems unlikely that a resident in cardiology resident in Brazil would be able to master the necessary knowledge and abilities in a 2-year training duration. As a result, there is an ongoing discussion in Brazil to have a single focused year of internal medicine training (instead of the current two years, as in France and Spain) before starting a new model of a 3-year cardiology residency.

## Required competencies and training schedule

To better understand how knowledge is acquired, one may also look at the core rotations trainees must complete. The government sector responsible for the coordination of medical training throughout Brazil is the Ministry of Education (with active participation of the Ministry of Health), and the CNRM.^17^ The CNRM was founded in 1977 and has made several improvements to the medical residency, as follow:

- Regulation of work hours: currently, a medical resident in Brazil (of any specialty) should work up to 60 hours per week, with no more than 24 hours of in-hospital shift activities (in-home on-call is not permitted);
- Wages and salaries: the resident doctor is paid monthly by the Government (Federal, State, or County) or by a private institution. All residents receive the same amount, regardless of the year of training or the field of training (currently there are 53 different medical specialties officially recognized in Brazil). Usually, no extra payment is granted for night shifts. Housing and food assistance are commonly offered, especially for those in greater need. Working at night shifts outside the residency program (“moonlighting”) and “locuming” are not prohibited, as long as they do not interfere with the residency program;
- Training supervision: the definition of residency is “training at service under supervision”, which means that the resident must be overseen by an attending physician at all time, including night, weekend and holiday shifts.

Once the CNRM requirements are fulfilled, each program has the flexibility to adapt the program according to the local reality. For cardiology, CNRM requires the trainee to spend at least half of the total training time in inpatient care, in the emergency department, wards or coronary care units (CCU). Around one-fifth of the training time must be dedicated to outpatient clinics, and at least 5% of the time should be spent learning diagnostic methods. Congenital heart disease and post-operative care are also considered mandatory rotations for all cardiology trainees.^18^ Importantly, as in North America and some European countries, cardiology residency programs in Brazil are planning to implement a “competency-based curriculum”. This approach is focused on evaluating the trainees according to specific learner outcomes, with emphasis on a formative, instead of a summative assessment, leaving behind the traditional time-based curriculum and passive learning methodology.^19^ The discussions are still ongoing, and it will probably take some time until the new proposal is fully implemented in Brazil.

In the USA, the Accreditation Council for Graduate Medical Education (ACGME) oversees training programs across the country and establishes general basic requirements for training sites and educators.^10^ The ACC also publishes the Core Cardiovascular Training Statement, currently in the fourth version (COCATS 4), with recommendations for levels of trainings and milestones within each component of the cardiovascular training.^14^ Both documents from the ACC and ACGME are aligned to and focused on competency-based learning. The general core competencies are: patient care; medical knowledge; practice-based learning and improvement; interpersonal communication skills; professionalism; and system-based practice.^14^ Of note, the Brazilian Society of Cardiology (BSC) also published a guideline for cardiovascular training in the Brazil.^20^

In Canada, cardiology training programs are supervised by the Royal College of Physicians and Surgeons of Canada. Along the three-year cardiology Residency Program, the minimum requirements comprehend: 15 training blocks of clinical residency (CCU, wards, consults, clinics), 15 blocks of laboratory-based residency (cardiac catheterization, electrophysiology, nuclear cardiology, echocardiography), two research blocks, and four blocks of electives.^21^ Additionally, clinical and academic contents of the program must fulfill all of the CanMEDS roles for the cardiology specialty. CanMEDS is a “framework that identifies and describes the abilities physicians require to effectively meet the healthcare needs of the people they serve”.^22^ According to CanMEDS, medical competencies are grouped under seven key roles: medical expert, communicator, collaborator, leader, health advocate, scholar and professional.^23^ Training programs are supposed to offer their trainees opportunities to master each one of these roles in the scope of practice. Canada is also moving towards implementing a competency-based medical education curriculum for all residency programs, through the “Competence by Design” initiative.^24^ This is based on milestones to be achieved as the resident advances through the training program, from the entrance in the residency program until the transition to the unsupervised medical practice. The target year for Canadian cardiology Residency programs to launch the “Competence by Design” is 2020. In Spain, trainees gain access to the cardiology Residency via the MIR test (“Médico Interno Residente”), right after leaving medical school, and the training lasts five years. In the first year, most of the rotations are usually related to internal medicine. In the second year, activities are divided between CCU, cardiology wards and consults. The third year is dedicated to non-invasive tests, such as echocardiography and cardiac stress tests. In the fourth year, around six months should be spent in the cardiac catheterization laboratory and 4 months in an electrophysiology service. The last year has a more flexible curriculum, at the discretion of each hospital. Residents may rotate on areas such as congenital heart disease and heart transplantation and/or can spend more time doing research and elective rotations.^25^

When compared to trainees from North America and some European countries, medical residents in Brazil spend less time in non-invasive tests and in the catheterization laboratory, since those abilities are developed in depth by those who choose to pursue further training in these subspecialties. Training in North America and Europe generally includes completing mandatory, procedural logs and documentation.^10,15,18^ In addition, in Brazil, little emphasis is given to research, contrary to countries with longer training length.

Residency training programs in Brazil are aimed at practical aspects of cardiology (around 80% of the time), and didactic activities, such as lectures, seminars, etc., are developed in the remaining 20% of total time. Again, changing the residency program duration from two to three years, would also contribute to a better training in important areas, such as research, among others.

## Availability and distribution of training centers

**Figure 1:**
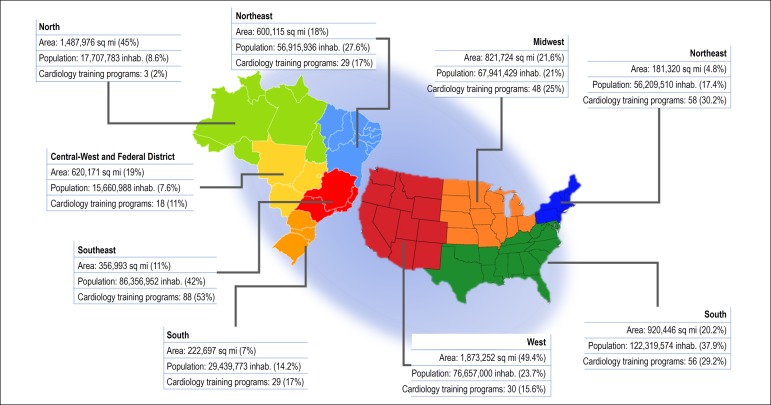
Comparison between Brazil and the USA (without Puerto Rico) regarding Cardiology Residency (Fellowship) Program distribution. Inhab.: inhabitants.^d^ ^27,28,30,35^

In Brazil, in 2017, 502 new residents^a^ started their training in 167 cardiology Programs, unevenly distributed throughout the regions of the country (average of 0.24 medical residents/100,000 inhabitants);^26^ the respective numbers for the USA (2016/2017 period) were 855 new cardiology Fellows in 193 Programs (0.26 new medical residents/100,000 inhabitants).^27^ However, the proportion of Cardiologists/100,000 inhabitants in both countries is, respectively, 7.47 for Brazil and 6.83 for the USA ^(^^26,28-30^^)^, ^b^ (Figure 1).

**Figure 2:**
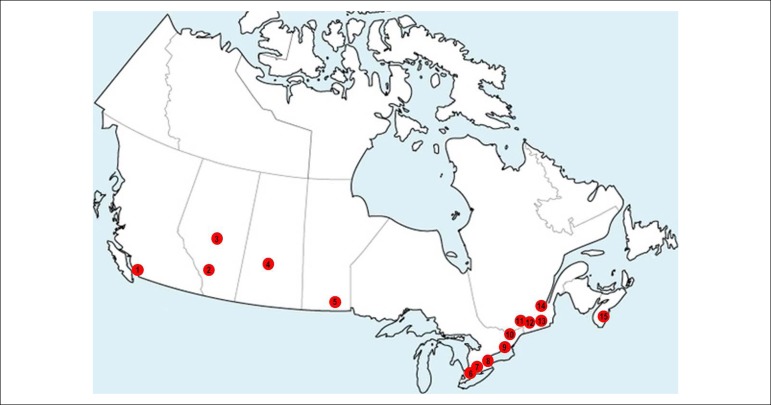
Geographical representation of all Cardiology training programs in Canada. 1: University of British Columbia (Vancouver, BC); 2: University of Calgary (Calgary, AB); 3: University of Alberta (Edmonton, AB); 4: University of Saskatchewan (Saskatoon, SK); 5: University of Manitoba (Winnipeg, MB); 6: University of Western Ontario (London, ON); 7: McMaster University (Hamilton, ON); 8: University of Toronto (Toronto, ON); 9: Queen’s University (Kingston, Ontario); 10: University of Ottawa (Ottawa, ON); 11: McGuill University (Montreal, QC); 12: Université de Montréal (Montreal, QC); 13: Université de Sherbrooke (Sherbrooke, QC); 14: Université Laval (Quebec City); 15: Dalhousie University (Halifax, NS).^31^

In Canada, there are 15 cardiology Residency Programs. A total of 58 new residents (PGY-4, postgraduate year four in the Canadian numerical scheme) started their training in Adult cardiology in the 2016/2017 period (0.16 new residents/100,000 inhabitants), most of them (50 residents) receiving regular government funding (Figure 2). Most of the programs are held in the provinces of Ontario (21 new cardiology residents within five programs), and Quebec (16 new cardiology residents within four programs), especially in the cities of Toronto and Montreal, respectively. Canada is a large country territory-wise, but with the vast majority of its 35 million inhabitants living within 100 miles of the USA border.^31,32^

Brazil also has significant variability in population density and financial resources across the country and, consequently, the distribution of the residency programs is very unequal, and most of the programs are held in institutions of the South and the Southeast regions (Figure 1). In fact, a single State out of the total 26, the São Paulo State, supports more than one-third of all available positions in the whole country. This reflects the distribution of the medical schools in these areas; the Southeast region concentrates most of the medical schools, while the North region, occupied mainly by the Amazon rainforest and with the smallest population density in the country, exhibits the smallest number of medical schools and cardiology Residency programs. In the USA, the distribution of the Residency Programs in cardiology is also unequal, but not like Brazil. Despite having the smallest geographical area, the US Northeast region houses the majority of positions, and the New York State alone concentrates approximately one-eighth of them all.^27^ Creating more training programs and facilities in more distant locations in Brazil could help improve cardiovascular care in the inner regions of the country. Local authorities have been working on this issue during the past years, but with limited success.

Board certification

Each country has a method to certify that a doctor is legally recognized as a specialist in a field. In the USA, the American Board of Internal Medicine is the agency responsible for offering physicians the certification in cardiovascular care. In Europe, each nation has its own agency, such as the Joint Royal Colleges of Physicians Training Board in the United Kingdom and the “College National des Enseignants de Cardiologie” in France. To date, there is not a single, unified European examination valid for all countries, although some initiatives have been proposed. In Brazil, after finishing the residency, the doctor is automatically certified by the CNRM and Federal Council of Medicine as a Cardiologist. Additionally, to be certified as a Cardiologist by the Brazilian Medical Association, the physician must apply for the BSC exam, which consists of a written examination, applied once a year, during the BSC National Congress. If approved, this professional will be certified by the BSC, Brazilian Medical Association and by the Federal Council of Medicine.

In Brazil, besides the residency programs supervised by the CNRM, there are 20 programs, accredited and supervised by the BSC (not by the CNRM) throughout the country.^c^ In general, the core curriculum is similar to the CNRM programs,^20^ but some differences should be noted: 1) in the BSC training programs no salary is paid to the trainee and 2) no automatic certification is granted by the Federal Council of Medicine; the trainees must undergo the BSC Board Certification test in order to be certified as a Cardiologist.

## Final comments

The model of the medical residency programs reflects the socioeconomic conditions, and the organization of the educational and health systems of each country. A limitation of the present text is the lack of data in the literature describing and comparing different cardiology training programs around the globe. This impairs our ability to make a more evidence-based comparison and many inferences presented in this manuscript are derived from the authors’ opinions and experiences. With these points in mind, in our understanding the main strengths of the cardiology Residency Programs in Brazil are: 1) one centralized national coordination (CNRM), responsible for the supervision and the rules that are valid for all programs; 2) the rigorous selection process candidates must go through to advance to the next level of training. On the other hand, this international perspective identifies opportunities for improvement, such as the fact that two years for training in General cardiology is likely too short, given the complexity of modern cardiology.

In countries like Brazil, with huge regional differences, it is imperative to make proposals for an equal provision of good Medicine all over the country. Yet, given the differences observed among the cardiology Residency Programs worldwide, it would be very useful if our professional governing bodies defined a minimum standardized curriculum for the training of new Cardiologists, considering the characteristics of the country. Possibly, a three-year residency, with a competency-based curriculum, offering a balanced amount of patient care, procedures and diagnostic test training, organized in time-limited rotations and longitudinal activities (such as an integrated outpatient clinic), and a time dedicated to research, would be a starting point for discussion about harmonization of the residency programs. Further, it would be important that medical societies across the world recognize these training differences, so that they could tailor educational programs in cardiology (including scientific meetings and conferences) for the needs of the developing world. Besides giving better care to our underserved population, these initiatives would facilitate collaboration and exchange experiences with cardiologists internationally.

In conclusion, the development of an international standardized minimum curriculum for the cardiology residency training programs, to be customized according to individual country characteristics, would be very useful and promote exchange of experience internationally. In our opinion, the cardiology training in Brazil needs to be improved based on the programs conducted in developed countries. In order to achieve this goal, it is necessary an urgent mobilization of different sectors of the cardiology community, such as the cardiology Residency programs, the BSC, and the CNRM, among others.
